# The relationship between PM2.5 and the onset and exacerbation of childhood asthma: a short communication

**DOI:** 10.3389/fped.2023.1191852

**Published:** 2023-08-01

**Authors:** Yue Zhang, Xixi Yin, Xiangrong Zheng

**Affiliations:** ^1^ Department of Pediatrics, Xiangya Hospital, Central South University, Changsha, China; ^2^ The Center of Respiratory Medicine, Xiangya Hospital, Central South University, Changsha, China

**Keywords:** asthma, PM2.5, children, asthma attack, air pollution

## Abstract

Much is known about the link between air pollution and asthma in adults, particularly fine particulate matter (PM2.5). Studies have found that certain levels of fine PM2.5 can increase airway responsiveness and worsen asthma. PM2.5 may play a role in the onset and exacerbation of childhood asthma. However, there is little in the literature on how PM2.5 affects asthma attacks and exacerbations in children. Asthma is a common chronic disease in children, and air pollution can aggravate it. The effect of PM2.5 on childhood asthma needs further research. By evaluating, reviewing, and collating existing results in this area, this paper aims to explore the relationship between PM2.5 and asthma onset and exacerbation in children.

## 1. Introduction

Asthma is a chronic inflammatory disease of the airways and is associated with bronchial hyperresponsiveness, reversible airflow limitation, recurrent wheezing, chest tightness, and coughing (1). Asthma is thought to be caused by a combination of genetic and external factors (2). In addition to individual genetic profiles, abnormal environmental stimuli, such as exposure to air pollution and allergens, and infection with respiratory viruses, are also important in the development of asthma (3).

Particulate matter 2.5 (PM2.5), also known as lung-penetrable particulate matter, refers to particulate matter with an aerodynamic diameter of not more than 2.5 μm in the atmosphere and is the main component in the atmosphere during hazy weather. PM2.5 is rich in toxic and harmful substances, which can threaten human health due to factors such as small particle size, efficient inhalation into the lungs, and its persistence in the atmosphere (4). PM2.5 can induce immune and inflammatory responses in the lungs, induce the release of related cytokines, lead to the occurrence of asthma-related cardiopulmonary diseases, and impact the incidence and mortality of cardiopulmonary diseases. There are many studies examining the impact of PM2.5 on the occurrence and development of asthma, but there is a lack of clear context in the field of childhood asthma. This paper aims to explore the relationship between PM2.5 and asthma onset and exacerbation in children.

## 2. Materials and methods

### 2.1. Literature search

Articles released between 2017 and 2022 were included in the search. Publications concerning asbestos, radon, methane, biological pollutants, and severe carbon monoxide exposure were excluded, and those that focused on exposure to fine particulate matter in the environment (specifically PM2.5) were included. The following online bibliographic database was used: PubMed. The search results are stored in the online reference manager, EndNote.

### 2.2. Paper screening and data extraction

Once the search was complete, titles and abstracts were screened based on inclusion criteria to identify relevant studies. In all cases, a conservative strategy was adopted, i.e., if the relevance or other aspects of the paper were not evident from the title/abstract, the paper was available for full-text scanning. Finally, a narrative synthesis of the literature that met the inclusion criteria was carried out.

## 3. Results

### 3.1. PM2.5 is related to the occurrence of asthma

#### 3.1.1. Molecular mechanism of leading asthma

PM2.5 refers to particulate matter in the atmosphere with an aerodynamic diameter of not more than 2.5 μm, also known as inhalable lung particulate matter, and is one of the main components of air pollution. Fine particulate matter (PM2.5) exposure is linked to lung inflammation and airway hyperresponsiveness (AHR).

**Figure 1:**
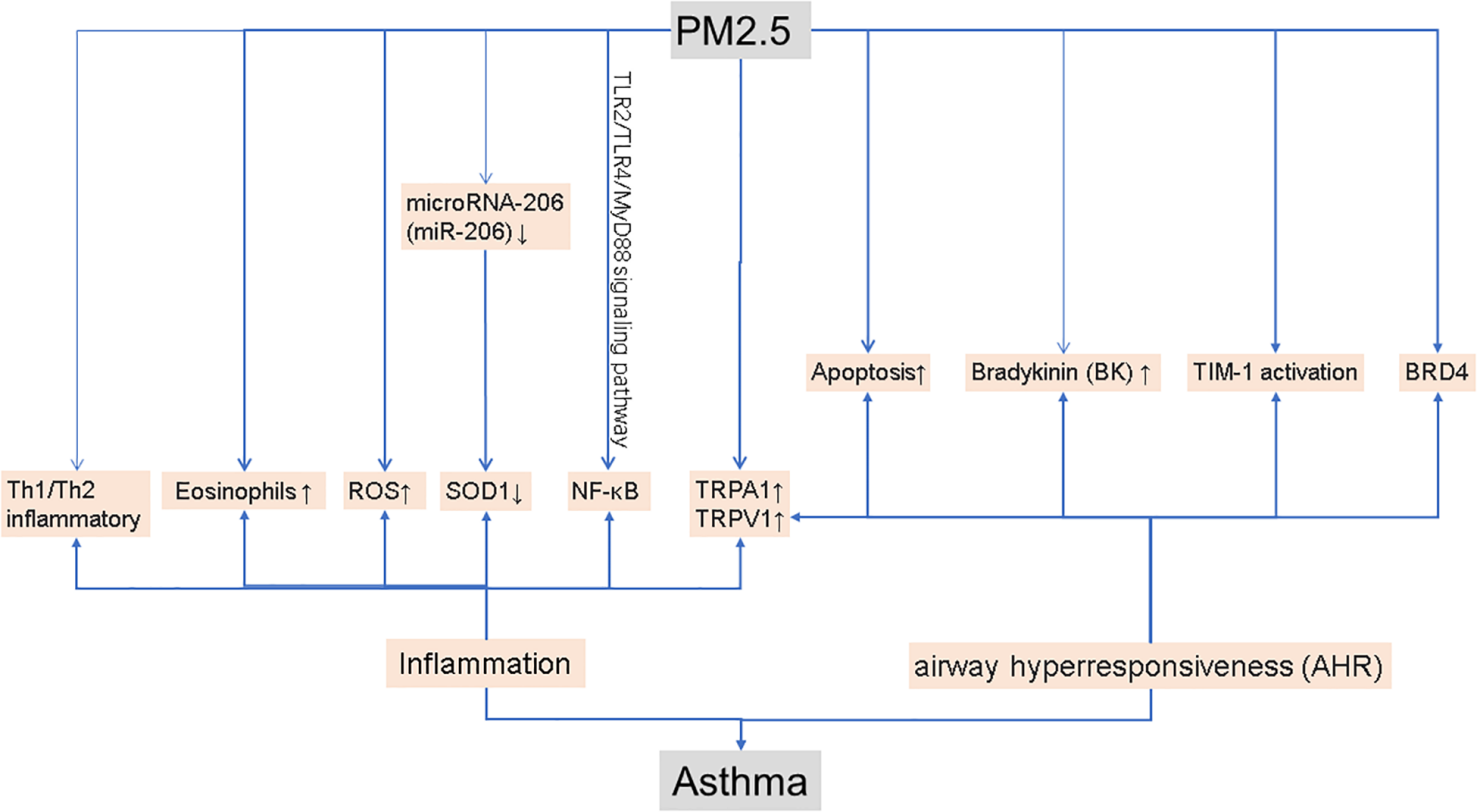
The molecular mechanism leading to asthma.

As shown in Figure 1, inflammation is one of the main mechanisms of asthma. In healthy mice, previous studies have shown that exposure to PM2.5 causes a mixed Th1/Th2 inflammatory response (5). In a mouse model of asthma, PM2.5 exposure causes allergic airway inflammation (6). Lesions in the lung increased after PM2.5 treatment, as did inflammation and EOS (eosinophils) in bronchoalveolar lavage fluid (BALF) was observed in ovalbumin (OVA) mice (7). In OVA-induced mice, PM2.5 may activate NF-κB to exacerbate allergic lung inflammation via the TLR2/TLR4/MyD88 signaling pathway (8). Airborne particulate matter (PM) from coal burning produces oxidative stress by increasing levels of reactive oxygen species (ROS), which is related to inflammatory responses in asthma. Scientists discovered that PM2.5 can increase the levels of antioxidant enzymes in the lung tissue of mice, as well as ROS levels (9). Furthermore, PM2.5 can induce the expression of microRNA-206 (miR-206) in mouse lung tissue. In asthmatic mice, miR-206 can target the 3′-UTR of SOD1 and inhibit its expression, aggravated lung inflammation and asthma symptoms (10).

AHR is also a major culprit in asthma. Studies have shown that extracellular vesicles (PM 2.5-EVs) released by PM 2.5-treated human bronchial epithelial (HBE) cells promote cytotoxicity in “horizontal” HBE cells and improve the contractility of “longitudinal” sensitive human bronchial smooth muscle cells (HBSMCs) (11). Increased apoptosis and TIM-1 activation are linked to PM2.5-induced AHR exacerbation in allergic asthma patients (12). According to a research study, PM2.5 can enhance airway hyperresponsiveness, which is triggered by necroptosis-related inflammation (13). BRD4 is a crucial component of PM2.5-induced AHR, which can be improved by inhibiting BRD4 (14). Researchers conducted a series of experiments by constructing a mouse model of asthma and discovered that PM2.5 can increase the expression of TRPA1 (transient receptor potential ankyrin 1) and TRPV1 (transient receptor potential vanilloid 1) in mouse lung tissue, and aggravates the effect of asthma in this animal model (15). TRPA1 and TRPV1 may both play a vital role in lung inflammation and AHR (16). In bronchopulmonary tissue, PM2.5 can stimulate kallikrein expression and the release of bradykinin (BK), and kallikrein inhibitors can reverse these alterations, demonstrating that kallikrein is relevant in PM2.5-induced airway hyperresponsiveness (17).

These findings could lead to new treatment targets for asthma exacerbations caused by PM2.5. The author suggests that analyzing the research of the above scholars shows that PM2.5 can trigger an inflammatory response and can also lead to airway barrier dysfunction, thereby inducing asthma.

#### 3.1.2. Epidemiological evidence that PM2.5 causes asthma

There are many published studies providing epidemiological evidence of the association between PM2.5 exposure and the occurrence and development of asthma. Air pollution, which includes NO_2_, PM2.5, and BC (black carbon), is responsible for nearly 48% of asthma cases in Barcelona each year (18). Children's wheezing and rhinitis may be exacerbated by ambient PM 2.5 (19). Long-term exposure to PM 2.5 has been linked to an increased risk of asthma in Chinese preschool children, according to surveys, and children who live in suburban or rural areas are much more likely to be exposed to PM2.5 (20). Researchers such as Rosalyn Singleton studied the housing characteristics and indoor air quality in the homes of Alaskan families with children with acute lung disease and found that high levels of PM2.5 were associated with respiratory symptoms in these children (21). Scholars such as Audrey Flak Pennington have also proven that there is a link between early-life exposure to air pollution from mobile sources and the incidence of childhood asthma (22).

Owing to inflammatory cell infiltration in the subcutaneous tissue of the airway, and pathological changes in airway architecture, outdoor coal burning (exposure to PM 2.5) may trigger airway inflammatory immune responses and exacerbate peribronchiolar inflammation. Stéphanie M. Holm and others have shown that cooking behavior may increase the burden of PM2.5 on children with asthma, which can lead to asthma symptoms (23). According to Hutchinson et al., who analyzed observational studies and two-way case-crossover analyses after the 2007 San Diego wildfires (where PM2.5 was significantly elevated), an increase in respiratory diagnoses, especially asthma, was found (24). Children aged 0–9 years had an increased risk of asthma and acute bronchitis during the wildfire (RR = 1.57, 95% CI: 1.21–2.02) and after the wildfire (RR = 2.11, 95% CI: 1.86–2.40). PM2.5 exposure from the wildfire has resulted in an increase in outpatient visits for respiratory illnesses, particularly among children (25).

PM2.5 can harm lung function. Using data from the Escort Intervention Study into the association between peak expiratory flow rate (PEFR) and indoor PM2.5 exposure levels in children with asthma, Kim et al. observed that increased indoor PM2.5 levels significantly reduced the PEFR (26). Higher PM2.5 concentrations can lead to decreased forced expiratory volume (FEV), forced expiratory volume in one second (FEV1), and maximum midexpiratory flow (MMEF) values (27). In addition, the research reported by Zhao et al. had similar findings, in which they noted that exposure to soluble PM2.5 extract can lead to airway barrier dysfunction (28).

The author suggests that the above research shows that PM2.5 has an impact on the occurrence and development of asthma, but because PM2.5 is not the only pollutant in ambient air, and other confounding factors, the impact of PM2.5 on asthma cannot be precisely defined.

### 3.2. PM2.5 leads to increased asthma-related doctor-seeking behavior in children

Several studies have found increased medical-seeking behavior among children with respiratory illnesses in areas with high air pollution. A study by Baek et al. reveals an association between ambient air pollution and hospital length of stay (LOS) for childhood asthma in South Texas. PM2.5 concentrations were positively correlated with LOS in children aged 5–11 years in an age-stratified model (29). Results from another of his studies indicate that short-term (4-day) exposure to air pollutants may increase the risk of preventable readmissions of pediatric asthma patients (30). A study by Davila Cordova and others into the association between PM2.5 concentration and outpatient visits at the Health Center for Respiratory Diseases for Children Under 5 in Lima, Peru, found that increased emissions of atmospheric pollutants such as PM2.5 may contribute to health centers experiencing an increased number of visits by children suffering from respiratory diseases (acute lower respiratory infections like bronchitis, pneumonia, and asthma) (31). Goodman and other scholars found that PM2.5 was significantly associated with an increased number of asthma hospitalizations in children aged 6–18 years (32). A study by Zafirah et al. found that exposure to PM2.5 was associated with asthma clinic visits in boys aged 0–18 years (33). Ma and Yu and their team researched the impact of particulate matter pollution on the number of children's asthma visits in Shanghai and found that this number was positively correlated with air pollutant concentrations and that PM2.5 had a significant impact on the number of children's asthma visits (34). Outdoor air pollution can lead to childhood asthma leading to emergency room visits and hospitalizations. The Air Quality Index (AQI) is associated with childhood asthma attacks, and the main pollutant affecting the AQI is PM2.5 (35). In Shijiazhuang City, there was a strong correlation between outdoor PM2.5 concentrations and hospital outpatient visits for children with respiratory problems (36).

According to research, exposure to air pollution may increase the chance of asthma attacks in children, putting them at a higher risk of hospitalization (37). According to research by AlBalawi et al., air pollution can exacerbate the symptoms of asthma patients. After mutual adjustment in a multipollutant model, the correlation between asthma-related emergency visits (AEDv) and PM2.5 stayed positive and statistically significant (38). In Shanghai, according to Liu et al., short-term exposure to ambient air pollutants raises the frequency of pediatric visits to the Asthma Emergency Department (ED) (39). According to the findings of Puvvula et al., exposure to air pollution mixtures (primarily PM2.5, pollen, and mold) can increase emergency room (ED) visits for children with asthma (40). To sum up, air pollution (particularly PM2.5) leads to an increase in the need for asthma-related healthcare, and PM2.5 has an impact on the development and aggravation of childhood asthma.

### 3.3. Improved air quality reduces asthma

Air pollution can lead to the occurrence and aggravation of asthma, and many researchers have found that improving air quality will decrease the occurrence of asthma. Children with acute respiratory infection (ARI) symptoms reported higher mean 1-year PM2.5 exposure than children without ARI symptoms. Long-term exposure to high PM2.5 levels in young children may increase the risk of acute respiratory illness (41). Garcia et al. found that reductions in ambient PM2.5 between 1993 and 2014 were significantly associated with lower asthma incidence (42). Cui et al. studied the association between filtering out bedroom particulate matter and airway pathophysiological changes in children with asthma, and the results showed that filtration of indoor PM2.5 can improve asthma symptoms (43). Using a high efficiency particulate air (HEPA) filter significantly reduced the levels of indoor traffic particulate matter and improved treatment outcomes, and the quality of life, of children with uncontrolled asthma (44). HEPA filtration can effectively lessen asthma rates in children and governments should make air purifiers readily available to the most susceptible low-income populations (45). This demonstrates that we have to improve air quality to prevent asthma attacks in children (46). Researchers noted that increasing the number of trees in areas improved local air quality and that asthma hospitalizations and emergency department visits were fewer, by looking at the data from hospitals and air quality data (PM2.5) (47). Due to improvements in ambient air pollution, the incidence rate and associated respiratory problems in school-aged youngsters in southern Taiwan has decreased (48). The US Regional Greenhouse Gas Initiative (RGGI) is beneficial to children's health (49). In short, we can assume that air pollution has an important impact on the occurrence and development of asthma and that improving the air quality can reduce the occurrence and development of the disease.

## 4. Conclusions

PM2.5 has an important role in the occurrence, development, and aggravation of childhood asthma. Epidemiological evidence is apparently sufficient, but research into the mechanism is still insufficient. In-depth research to uncover the mechanism is necessary for the treatment and prevention of asthma. The literature reviewed in this study is from the past 5 years, which should reflect the latest progress in this field. As economies develop, an increase in air pollution is almost inevitable, so research into the influence of PM2.5 on the occurrence and development of childhood asthma is necessary. Raising environmental health awareness and implementing effective measures to improve air quality will benefit children's health. Extensive research has focused on the impact of PM2.5 on the occurrence and development of childhood asthma, but these studies still fall short of a complete understanding of the mechanisms elucidating how PM2.5 causes and exacerbates the disease. The author hopes that scholars in the future will pursue this knowledge.

## Data Availability

The original contributions presented in the study are included in the article/Supplementary Material, further inquiries can be directed to the corresponding author.
